# Normative physical performance values and sex-bases developmental trajectories in Mexican school sport programs: a longitudinal study using VALD technology

**DOI:** 10.3389/fspor.2026.1825451

**Published:** 2026-06-03

**Authors:** Luis Alberto Dueñas-Dorado, Irvin Jesé Gaona-Carranza, Pablo Tadeo Ríos-Gallardo, Raquel Morquecho-Sánchez, Renato Treviño Theriot, José Alberto Pérez-García, Mireya Medina Villanueva, Rubén Ramírez-Nava

**Affiliations:** 1 Faculty of Sports Organization, Universidad Autónoma de Nuevo León, San Nicolás de los Garza, Mexico; 2 Affinitas Education, London, United Kingdom; 3 VALD Performance, Brisbane, QLD, Australia

**Keywords:** benchmarking, change-of-direction, change-of-direction ability, countermovement jump, Latin America, normative values, physical development, physical education

## Abstract

The purpose of this study was to establish normative values and analyze the longitudinal development of physical performance in students from Mexican private institutions using VALD technology. Data were collected from 2,473 students aged 5 to 16 years who completed standardized tests of strength, power, speed, and agility. Two-way ANOVA revealed significant main effects of age and sex (all *p* < .001) and significant age-by-sex interaction effects across all tests, indicating that performance trajectories diverge between sexes with increasing age, particularly from age 12 onward. Centile curves were generated using generalized additive models to serve as normative references for school sport management. These findings provide the first technology-validated normative physical performance dataset for a school-aged Latin American population and offer a practical benchmarking framework for evidence-based planning in educational sport programs.

## 1 Introduction

Objective monitoring of physical performance in school-aged populations has gained considerable traction as a tool for supporting talent development, health surveillance, and evidence-based pedagogical decision-making in educational sport programs (1, 2). The integration of validated assessment technologies (particularly force plate and timing systems) enables the collection of reliable, continuous performance data that can inform multi-year program planning, individualized training, and institutional benchmarking (3). Within this framework, normative reference values play a central role: they allow practitioners to locate individual or group performance within a population distribution, identify developmental outliers, and evaluate program effectiveness against objective standards (4, 5).

Despite the growing availability of normative physical fitness data in European and North American youth populations, as reported by Mascherini et al. (6), Nowak et al. (7)**,** and Taylor et al. (8), no technology-validated normative dataset currently exists for school-aged children and adolescents in Latin America. Existing regional studies are predominantly cross-sectional, rely on field-based fitness tests without instrumented measurement, and were conducted outside the 5–16 year age range of greatest developmental interest for school sport programs. This evidence gap limits the applicability of international references to Mexican and broader Latin American school populations, where physical development profiles may differ due to distinct nutritional, socioeconomic, and sport participation patterns. The absence of longitudinal normative data further constrains the ability of school sport coordinators to establish realistic age-specific benchmarks or evaluate program effectiveness over time.

Private educational institutions in Mexico represent a feasibility setting for technology-based longitudinal assessment: their organizational autonomy and resource infrastructure support the implementation of validated measurement systems such as VALD Performance (ForceDecks FD4000), which integrates hardware and software capable of quantifying key biomechanical variables (including jump height, force production, and sprint time) with high inter-session reliability (ICC = 0.91–0.99) (9). By accumulating multi-year performance data across four schools in the metropolitan area of Monterrey, this study generates a replicable model of institutional benchmarking applicable to the broader Mexican school sport context.

The primary aim of this study was to establish age- and sex-specific normative physical performance values and analyze longitudinal developmental trajectories in students from Mexican private institutions using VALD technology, with the purpose of generating a benchmarking framework applicable to school sport management. The specific objectives were to: (a) describe the developmental evolution of strength, power, speed, and agility by age and sex; (b) quantify the magnitude and age at onset of sex-based performance divergence; (c) generate centile curves as normative reference standards; and (d) examine the predictive utility of age for physical performance using linear regression. We hypothesized that: (H1) power test performance (CMJ, SJ) would show significant non-linear improvement with age; (H2) speed and agility performance would improve progressively with age across both sexes; (H3) a large sex-based performance gap (Cohen's d > 0.8) would emerge from approximately age 12–14 onward; and (H4) age would explain a substantial proportion of performance variance (R^2^ > 0.60) across all tests.

## 2 Materials and methods

### 2.1 Study design

A longitudinal descriptive-comparative study with a quantitative and inferential approach was conducted between 2022 and 2024 across four private schools in the metropolitan area of Monterrey, Mexico. All participating institutions follow the national curriculum mandated by the Secretariat of Public Education (SEP), which requires a minimum of two physical education sessions per week (50 min each) across all grade levels. In addition, all four schools operate structured extracurricular sport programs (including football, basketball, volleyball, and athletics) with training frequencies ranging from two to four sessions per week depending on grade level and competitive season. Students participated in assessments as part of the institutional physical evaluation programme; they were not pre-selected based on athletic ability or sport specialization. Physical performance records were collected using VALD instruments and encompassed five standardized physical tests.

### 2.2 Participants

A total of 2,658 student records were initially retrieved from four participating private schools. After removing records with invalid or implausible demographic data and entries outside the target age range (5–16 years), 2,512 unique students were identified as the enrolled assessment population. Of these, 2,473 students (1,272 males; 1,201 females) completed at least one valid performance test and constitute the final analytical sample reported throughout this manuscript. The 39-student difference corresponds to individuals absent on testing days or whose records could not be matched to performance data files. Inclusion criteria were: (a) parental informed consent provided by legal guardians; (b) absence of musculoskeletal injuries at the time of testing; and (c) completion of at least one valid test trial. The study was approved by the Ethics Committee of the Faculty of Sports Organization, Universidad Autónoma de Nuevo León (protocol: UANL-2024-045-FOD), and conducted in accordance with the Declaration of Helsinki (2013 revision).

All participants were enrolled in the physical education curriculum of their respective institutions, which included a minimum of two structured physical education sessions per week (90 min total) aligned with the Mexican national curriculum for basic and middle education (SEP guidelines). Sessions emphasized fundamental movement skills, sport-specific activities, and recreational games appropriate to each developmental stage. No participants were enrolled in formal extracurricular athletic training programs at the time of baseline assessment, though sport participation outside school hours was not systematically controlled.

### 2.3 Instruments and physical tests

VALD Performance systems (ForceDecks FD4000, VALD Performance, Brisbane, Australia) operating at a sampling frequency of 1,000 Hz were used. The system integrates dual piezoelectric force plates and was zero-calibrated before each testing session according to the manufacturer's specifications (10), demonstrating high inter-session reliability (Intraclass Correlation Coefficient, ICC = 0.91–0.99) for all reported metrics in youth athlete populations (9). The following five standardized tests were administered:

1. Countermovement Jump (CMJ): three attempts, 90 s rest, best trial retained, valid trial required bilateral foot contact and no arm swing;
2. Squat Jump (SJ): three attempts, same rest protocol, static start position, trials with negative impulse > 5 N·s discarded;
3. Handgrip Strength: bilateral measurement, two attempts per hand, maximum retained;
4. 20-m Sprint: single timed trial with familiarization, electronic timing;
5. T-Test Agility: single timed trial with practice repetition per Pauole et al. (11). All tests followed NSCA standardized protocols.

### 2.4 Procedure

Assessments were conducted in the school gymnasium of each institution with wooden flooring under standardized conditions. All participants wore athletic footwear. A familiarization session of one practice trial per test was provided before recorded attempts. Testing was conducted in groups of 8–12 students supervised by trained, VALD-certified personnel. Testing order was fixed across all schools: CMJ → SJ → Handgrip → 20-m Sprint → T-Test, with 3 min of seated rest between modalities. Data were stored in VALD Hub, exported in CSV format, and processed in R (v4.3.2; R Core Team, 2023).

### 2.5 Statistical analysis

Data are presented as mean ± SD unless otherwise stated. Descriptive statistics and percentile values were calculated for each age-sex stratum at the 5th, 25th, 50th, 75th, and 95th centiles, following the convention for youth physical fitness normative references. Each band serves a specific diagnostic function: P5 (lowest performance risk), P25 (below average), P50 (benchmarking anchor), P75 (above average), P95 (talent identification tier). Two-way ANOVA with age and sex as fixed factors tested for main effects and interactions across all metrics. Sex-based effect sizes within age groups were quantified using Cohen's d (small: 0.2; medium: 0.5; large: 0.8). Smoothed centile curves were generated using quantile Generalized Additive Models (GAMLSS). Simple linear regression models stratified by sex quantified average annual performance change (R^2^). Statistical significance was set at *α* = 0.05. All analyses were conducted in R v4.3.2 (RRID: SCR_001905) and RStudio (RRID: SCR_000432).

## 3 Results

The final analytical sample comprised 2,473 students (1,272 males; 1,201 females) aged 5–16 years. The complete performance dataset includes 9,042 valid CMJ records, 4,772 SJ records, 2,807 handgrip strength assessments, 2,310 20-m sprint trials, and 2,255 T-Test agility trials. The higher CMJ record count reflects repeated annual assessments across the 2022–2024 study period (Table 1).

**Table 1:** Participant demographics by age and gender.

Total	Age (years)	FEMALE	MALE
	5	1	0
	6	21	11
	7	107	102
	8	102	122
	9	124	123
	10	120	127
	11	116	132
	12	113	139
	13	123	126
	14	124	133
	15	135	144
	16	115	113
Total	—	1,201	1,272

**Figure 1:**
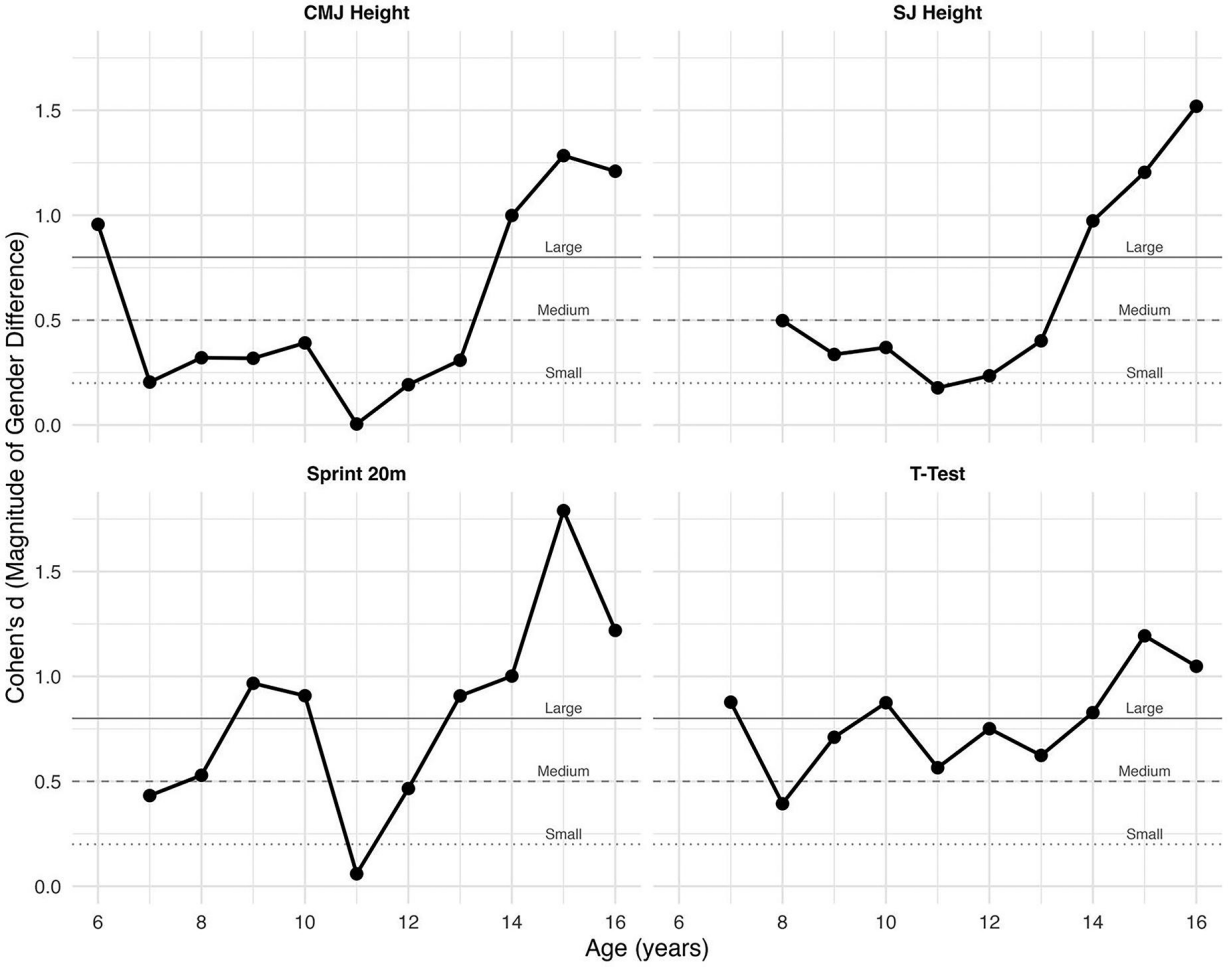
Distribution of Key physical performance metrics by age and sex. Boxplots illustrate the distribution of Countermovement Jump (CMJ) height, Squat Jump (SJ) height, 20-meter sprint time, and T-Test agility time across age groups, stratified by sex (female: Salmon; male: teal). The central line represents the median; the box indicates the interquartile range (25th to 75th percentile); whiskers extend to 1.5 times the interquartile range; points beyond the whiskers represent outliers.

 Figure 1 presents the distribution of performance for the four key physical assessments, stratified by age and sex. A clear developmental trend was evident across all metrics. Both Countermovement Jump (CMJ) and Squat Jump (SJ) height showed progressive improvement with increasing age for males and females. A sex-based performance difference emerged from approximately age 12 onward for jump tests and was apparent from early ages for speed and agility metrics, consistent with differential physical development profiles documented in the broader youth sport science literature. For time-based metrics, 20-m sprint and T-Test times decreased consistently with increasing age, reflecting progressive improvements in linear speed and change-of-direction ability.

CMJ normative values are presented in Table 2. The median (P50) jump height for males increased progressively from 9.90 cm at age 6 to 27.50 cm at age 15. A similar developmental trajectory was observed for females, whose median performance rose from 12.10 cm at age 5 to a peak of 20.30 cm at age 14. A notable performance divergence between sexes emerged from approximately age 12, consistent with age-associated changes in body composition and neuromuscular capacity documented in the literature for this developmental stage, although biological maturation was not directly assessed in this study. At age 16, mean CMJ height was 26.96 cm (SD = 6.81) for males and 19.76 cm (SD = 4.94) for females. Standard deviations and the widening gap between P5 and P95 with increasing age reflect greater intra-group performance variability in older cohorts, a typical finding in developmental biomechanics.

**Table 2:** Normative values for Countermovement Jump (CMJ) height (cm).

Age	n	Mean	SD	Percentiles				
				P5	P25	P50	P75	P95
FEMALE								
5	3	12.73	1.55	11.65	11.85	12.10	13.30	14.26
6	22	13.02	3.92	6.62	11.22	12.70	15.50	18.30
7	95	13.89	3.51	8.27	11.30	14.00	16.20	19.20
8	151	14.55	3.65	8.60	12.20	14.80	16.80	21.30
9	231	15.50	3.59	9.80	13.20	15.40	17.70	21.50
10	227	16.74	3.77	10.20	14.50	17.10	19.20	22.90
11	225	18.27	3.87	11.72	15.50	18.10	21.00	24.96
12	151	18.74	4.32	11.25	16.15	19.00	21.80	25.00
13	193	20.21	4.95	12.88	16.80	20.00	23.50	27.66
14	214	20.31	5.17	12.00	16.85	20.30	23.50	28.80
15	191	19.60	5.79	12.40	15.90	19.40	22.00	27.40
16	148	19.76	4.94	12.63	16.55	19.40	22.95	27.80
MALE								
6	11	9.74	2.87	6.30	7.45	9.90	10.80	14.35
7	74	14.69	4.30	7.83	11.90	14.60	17.65	21.74
8	224	15.80	4.16	9.13	13.00	15.90	18.50	21.94
9	201	16.76	4.27	10.70	13.80	16.80	19.20	23.30
10	235	18.28	4.10	11.30	15.80	18.50	21.00	24.26
11	256	18.30	4.59	12.17	15.40	17.60	20.80	26.30
12	266	19.60	4.58	12.83	16.60	19.35	22.65	27.30
13	212	21.88	5.81	12.71	18.30	21.50	25.97	32.10
14	221	25.78	5.78	16.10	21.40	26.30	29.90	34.70
15	204	27.46	6.44	17.53	23.70	27.50	31.42	37.59
16	120	26.96	6.81	15.86	22.78	27.30	32.23	37.10

Squat Jump (SJ) normative values are presented in Table 3. Male SJ height showed consistent improvement from 16.48 cm (SD = 4.00) at age 8 to 26.18 cm (SD = 5.72) at age 16. In contrast, female SJ height increased steadily to a peak of 18.97 cm (SD = 4.53) at age 14, followed by a performance plateau at ages 15 (18.52 cm) and 16 (18.24 cm), resulting in a sex-based gap of more than 43% at age 16. A comparable plateau in female jump performance during late adolescence has been reported in English school populations (8). These differences likely reflect differential developmental trajectories of lean muscle mass and neuromuscular drive between sexes, though causal attribution requires maturation data not collected in this study.

**Table 3:** Normative values for Squat Jump (SJ) height (cm).

Age	n	mean	sd	Percentiles						p90
				p05	P10	p25	p50_median	p75	p95	
FEMALE										
5	1	15.00	NA	15.00	15.00	15.00	15.00	15.00	15.00	15.00
6	2	16.45	1.77	15.33	15.45	15.82	16.45	17.07	17.57	17.45
7	7	14.70	5.35	8.01	8.82	10.75	15.90	17.90	21.18	19.86
8	51	14.48	4.04	8.60	9.90	11.30	14.20	17.10	20.70	18.80
9	53	15.10	3.61	9.90	11.04	12.40	15.20	17.10	20.04	18.80
10	82	16.65	3.86	11.02	11.83	14.22	16.60	19.40	22.68	21.58
11	67	17.06	3.42	11.96	12.60	14.70	17.10	19.30	23.10	21.08
12	71	17.79	3.76	11.35	12.60	15.10	17.90	20.60	23.10	22.40
13	46	17.67	5.86	10.60	11.95	13.20	16.05	20.70	28.47	24.15
14	63	18.97	4.53	11.41	12.96	16.15	19.00	22.60	25.70	25.46
15	85	18.52	6.29	10.90	12.10	14.30	17.10	21.60	29.20	25.50
16	90	18.24	4.68	11.47	12.20	14.85	17.80	21.00	26.42	24.24
MALE										
7	1	13.30	NA	13.30	13.30	13.30	13.30	13.30	13.30	13.30
8	80	16.48	4.00	10.30	16.25	13.80	16.25	19.45	22.15	20.22
9	58	16.38	3.97	10.30	16.40	14.20	16.40	18.82	22.85	21.58
10	80	18.08	3.85	11.30	18.10	15.85	18.10	20.30	24.23	22.50
11	103	17.74	4.16	11.83	17.90	13.80	17.90	20.10	25.45	23.42
12	116	18.76	4.45	11.28	18.60	15.85	18.60	21.20	26.80	24.70
13	146	19.98	5.66	11.90	19.40	16.12	19.40	23.90	29.60	27.20
14	173	23.87	5.51	13.60	24.60	19.80	24.60	28.00	31.76	29.68
15	162	25.91	5.97	17.30	25.55	22.05	25.55	29.20	36.80	33.37
16	68	26.18	5.72	16.71	26.15	22.15	26.15	30.02	36.00	33.91

Normative values for maximum handgrip strength are presented in Table 4. A strong positive association between age and grip strength was observed for both sexes (12). Females exhibited slightly higher mean strength than males at age 6 (76.08 N vs. 66.00 N), with comparable values until approximately age 9, after which a progressive male advantage emerged. Median male strength increased from 85.5 N at age 8 to 276.0 N at age 16, representing a more than threefold increase. Female median strength increased from 85.0 N to 194.0 N over the same period. Standard deviations increased substantially with age for both sexes, indicating greater heterogeneity in strength capabilities among older adolescents.

**Table 4:** Normative values for Maximum handgrip strength (N).

Age	n	mean	sd	Percentiles						
				p05	p10	p25	p50_median	p75	p90	p95
FEMALE										
5	1	99.00	NA	99.00	99.00	99.00	99.00	99.00	99.00	99.00
6	12	76.08	43.80	40.85	44.60	50.00	66.00	78.75	97.80	144.80
7	47	75.04	20.31	46.60	50.00	58.50	75.00	88.00	96.00	105.70
8	73	87.86	25.10	56.20	61.20	73.00	85.00	98.00	110.80	133.80
9	112	100.96	22.56	68.00	73.00	85.75	98.50	117.00	129.90	143.00
10	110	115.33	25.87	72.45	79.90	102.00	117.00	130.00	143.00	152.55
11	110	141.21	40.37	81.90	103.50	114.00	142.00	163.00	181.10	190.55
12	83	151.58	37.18	96.00	105.20	124.50	150.00	179.00	204.60	209.80
13	79	170.99	36.98	111.80	121.80	144.00	171.00	193.00	219.00	229.30
14	82	191.02	35.95	131.15	149.40	170.25	190.50	214.75	236.70	244.95
15	110	190.71	42.14	127.90	136.00	167.25	187.50	212.75	250.00	267.00
16	56	200.29	43.54	134.75	147.00	171.00	194.00	225.25	255.00	264.25
MALE										
6	5	66.00	16.70	43.40	49.80	69.00	70.00	75.00	77.40	78.20
7	26	84.38	21.92	55.25	56.50	68.00	78.50	104.25	116.00	116.00
8	104	92.12	33.70	55.45	69.00	79.75	85.50	102.00	112.70	122.00
9	115	109.66	22.72	77.40	81.00	94.00	108.00	123.50	141.20	152.00
10	119	127.72	35.76	73.90	83.80	101.00	130.00	151.00	160.00	169.30
11	143	128.40	46.80	84.00	84.00	84.00	119.00	153.50	178.40	209.60
12	125	151.59	39.80	83.00	105.40	125.00	150.00	174.00	210.00	222.80
13	144	197.02	53.90	120.05	138.30	158.75	190.50	227.50	264.40	279.70
14	117	229.26	59.94	146.00	164.80	188.00	221.00	269.00	299.40	311.80
15	109	273.12	65.07	175.80	185.40	230.00	269.00	316.00	356.80	381.20
16	76	266.79	53.86	166.75	190.50	225.75	276.00	301.50	337.50	351.75

Normative values for 20-m sprint performance are presented in Table 5. Mean sprint time decreased from 4.79 s (SD = 0.60) at age 7 to 3.30 s (SD = 0.33) at age 16 for males, and from 5.00 s (SD = 0.36) at age 7 to 3.75 s (SD = 0.40) at age 16 for females. The sex-based performance difference widened from approximately 0.25 s at age 8 to 0.45 s at age 16, while intra-group variability remained relatively stable across age cohorts. T-Test agility normative values are presented in Table 6. Females demonstrated superior agility at age 7 (mean = 20.47 s) compared with males (mean = 23.31 s); this advantage had dissipated by age 8, and from age 9 onward males consistently recorded faster times, with the performance gap continuing to widen through age 16. Consistent with the SJ data, female agility performance appeared to plateau after age 14. This early female advantage in agility, followed by progressive male superiority, is consistent with age-associated patterns of neuromotor development documented in the youth sport science literature, though direct maturation assessment was not performed.

**Table 5:** Normative values for 20-meter sprint time (s).

Age	n	mean	sd	Percentiles						
				p05	p10	p25	p50_median	p75	p90	p95
FEMALE										
6	1	4.01	NA	4.01	4.01	4.01	4.01	4.01	4.01	4.01
7	6	5.00	0.36	4.62	4.66	4.78	4.99	5.10	5.36	5.48
8	33	4.77	0.46	4.14	4.28	4.49	4.65	4.99	5.37	5.56
9	28	4.80	0.48	4.15	4.35	4.46	4.70	5.03	5.45	5.65
10	70	4.37	0.52	3.72	3.82	3.97	4.25	4.67	4.89	5.06
11	52	4.05	0.40	3.56	3.59	3.76	3.97	4.31	4.56	4.80
12	37	4.10	0.47	3.48	3.64	3.75	3.99	4.43	4.88	4.95
13	23	4.08	0.59	3.29	3.36	3.70	4.00	4.38	4.65	5.24
14	30	3.67	0.45	3.10	3.18	3.43	3.59	3.85	4.21	4.28
15	46	4.02	0.51	3.51	3.52	3.69	3.88	4.18	4.69	4.96
16	53	3.75	0.40	3.23	3.29	3.50	3.66	3.97	4.26	4.56
MALE										
7	9	4.79	0.60	4.16	4.38	4.54	4.73	4.93	5.19	5.69
8	64	4.52	0.48	3.94	4.13	4.27	4.40	4.81	5.17	5.29
9	49	4.31	0.52	3.59	3.81	3.91	4.23	4.59	4.95	5.05
10	74	3.96	0.36	3.54	3.57	3.71	3.93	4.07	4.44	4.60
11	89	4.07	0.33	3.49	3.58	3.78	4.23	4.35	4.35	4.48
12	66	3.89	0.43	3.14	3.31	3.60	3.94	4.16	4.32	4.48
13	72	3.59	0.46	2.94	3.08	3.24	3.55	3.85	4.11	4.48
14	85	3.29	0.29	2.91	2.99	3.06	3.21	3.45	3.75	3.87
15	89	3.24	0.34	2.79	2.90	3.02	3.17	3.44	3.63	3.82
16	49	3.30	0.33	2.92	2.95	3.08	3.28	3.41	3.65	3.83

**Table 6:** Normative values for T-Test time (s).

Age	n	mean	sd	Percentiles						
				p05	p10	p25	p50_median	p75	p90	p95
FEMALE										
6	2	14.76	3.16	12.75	12.97	13.64	14.76	15.88	16.55	16.77
7	5	20.47	2.23	17.99	18.18	18.74	20.41	22.68	22.69	22.70
8	34	19.33	2.23	15.61	16.72	18.08	19.16	21.15	21.91	22.71
9	32	19.02	3.30	15.35	15.68	17.33	18.52	19.85	20.61	24.19
10	75	16.12	2.22	12.93	13.27	14.38	15.98	17.83	18.79	19.22
11	63	14.77	1.92	12.29	12.90	13.64	14.37	15.37	17.06	17.62
12	37	14.59	1.56	12.38	12.85	13.35	14.33	16.06	16.85	16.90
13	24	15.03	2.92	11.62	12.36	12.81	14.46	16.81	19.42	19.98
14	27	13.32	1.76	11.27	11.38	11.95	13.04	14.27	15.67	16.42
15	54	14.98	3.44	11.32	11.54	13.16	14.27	16.39	17.83	19.13
16	39	13.96	1.75	11.33	11.60	12.80	14.13	15.42	16.21	16.62
MALE										
7	6	23.31	4.00	18.45	18.95	20.41	23.61	26.63	27.36	27.53
8	65	18.28	3.04	14.30	15.22	16.91	17.67	19.19	22.93	23.67
9	47	16.86	2.75	12.65	13.78	14.93	16.39	19.03	19.77	21.59
10	64	14.31	1.90	12.19	12.49	12.96	13.65	15.46	16.70	17.98
11	80	15.92	2.16	12.41	12.82	13.97	16.36	17.57	17.70	17.74
12	66	13.39	1.64	11.06	11.59	12.27	13.07	14.38	15.11	16.48
13	83	13.53	1.78	11.35	11.80	12.34	13.29	14.48	15.03	16.43
14	89	12.07	1.19	10.69	10.87	11.22	11.81	12.46	14.00	14.69
15	78	11.92	1.15	10.30	10.60	11.18	11.73	12.59	13.31	13.73
16	41	12.29	1.43	10.58	10.79	11.38	12.17	12.81	13.94	14.98

**Figure 2:**
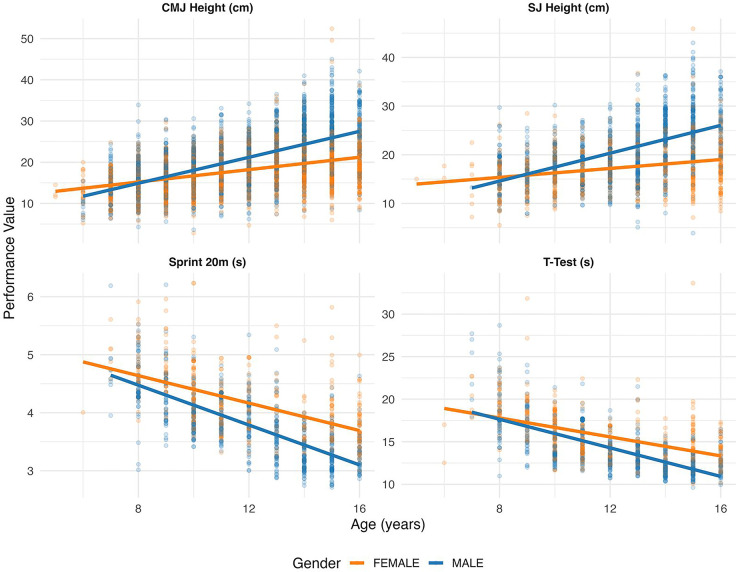
Age-specific percentile curves for physical performance tests by sex. Smoothed centile curves (P5, P25, P50, P75, P95) are presented separately for females (left column) and males (right column) across four performance metrics: CMJ height (cm), SJ height (cm), 20-meter sprint time (s), and T-Test agility time (s). Curves were generated using quantile Generalized Additive Models (GAMLSS). For time-based metrics, lower values indicate superior performance. The P50 (solid line) represents the normative median reference; P5 and P95 (long-dashed lines) define the lower and upper performance boundaries; P25 and P75 (dotted lines) indicate the interquartile normative range.

To provide a comprehensive visualization of the normative developmental trajectories, smoothed centile curves were generated for each performance metric using quantile Generalized Additive Models (GAMLSS), stratified by sex (Figure 2). For power-based tests (CMJ and SJ height), the male centile curves show a steep, sustained increase throughout the study period, while the female curves exhibit a clear deceleration approaching a performance plateau in late adolescence (approximately ages 14–16). For time-based tests (20-m sprint and T-Test), the curves illustrate rapid improvement across childhood and early adolescence for both sexes, with the male P50 curve continuing on a steeper downward trajectory than the female curve in the later teenage years. These centile charts provide a practical visual tool for locating individual student performance within age- and sex-specific normative distributions.

**Figure 3:**
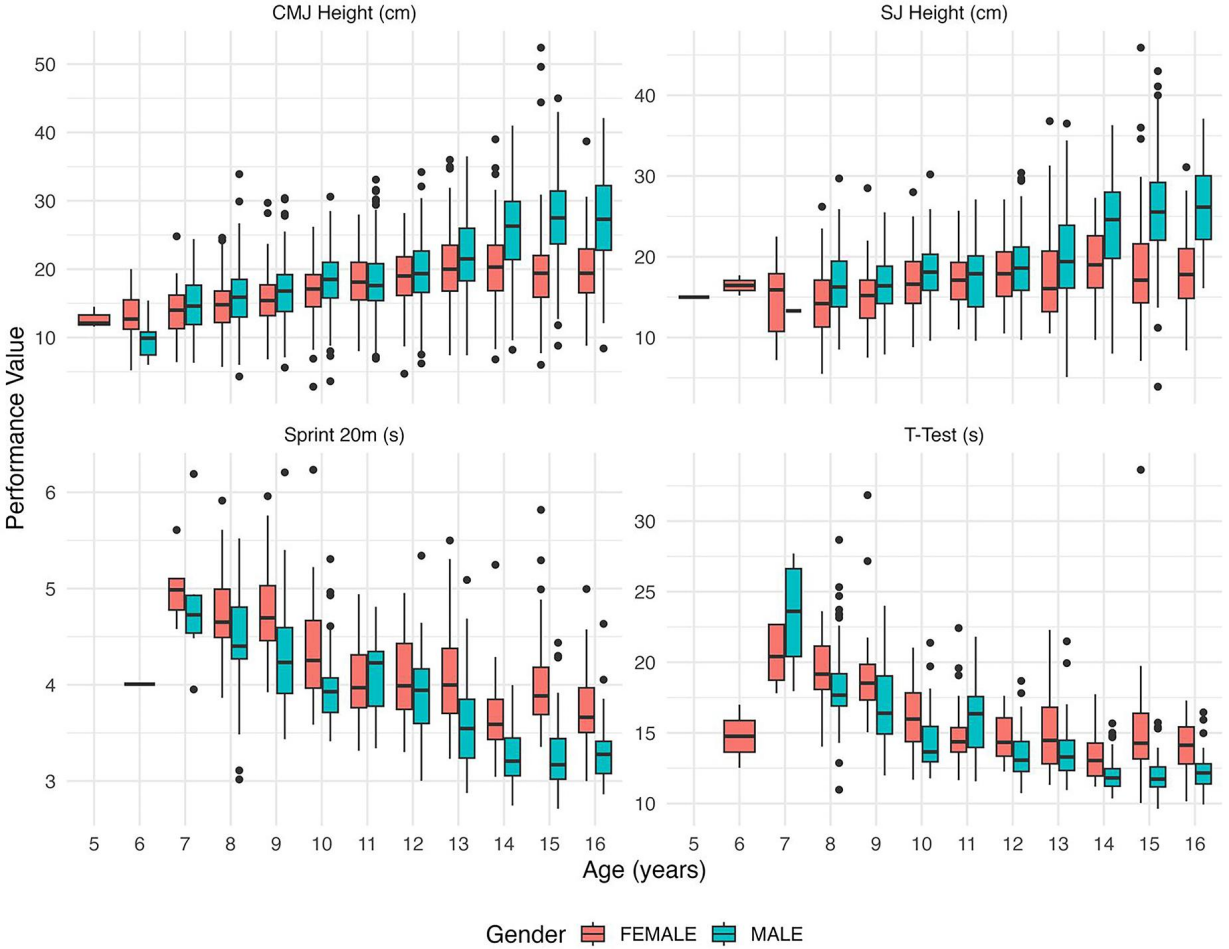
Developmental trajectory of sex-based performance differences (Cohen's d) Across age groups. Each point represents the absolute Cohen's d effect size for the sex-based difference in performance at each age group across four metrics: CMJ height, SJ height, 20-meter sprint time, and T-Test agility time. Horizontal reference lines indicate conventional effect size thresholds: small (d = 0.2, dotted), medium (d = 0.5, dashed), and large (d = 0.8, solid).

Two-way ANOVA confirmed significant main effects of age on all performance measures: CMJ height [F(11, 3,852) = 148.01, *p* < .001], SJ height [F(11, 1,583) = 45.44, *p* < .001], 20-m sprint time [F(10, 1,004) = 94.05, *p* < .001], and T-Test time [F(10, 990) = 91.87, *p* < .001]. Significant main effects of sex were also confirmed for all tests (CMJ: F(1, 3,852) = 294.46, *p* < .001; SJ: F(1, 1,583) = 181.38, *p* < .001; Sprint: F(1, 1,004) = 174.52, *p* < .001; T-Test: F(1, 990) = 80.34, *p* < .001). Significant age-by-sex interaction effects were present for all four metrics (all *p* < .001), providing statistical evidence that developmental trajectories differ between sexes across the studied age range. To quantify the magnitude of sex-based differences at each age, Cohen's d effect sizes were calculated and are presented in Figure 3. Effect sizes were small to negligible during middle childhood (approximately ages 7–12) for power-based tests. A large effect (d > 0.8) emerged from age 14 for CMJ and SJ, increasing further in late adolescence. For speed and agility, a small-to-medium sex-based difference was present from early ages, also amplifying to a large effect during the adolescent years. This developmental pattern is consistent with age-associated changes in strength, power, and body composition reported in youth populations; however, direct maturation assessment was not conducted in this study.

**Figure 4:**
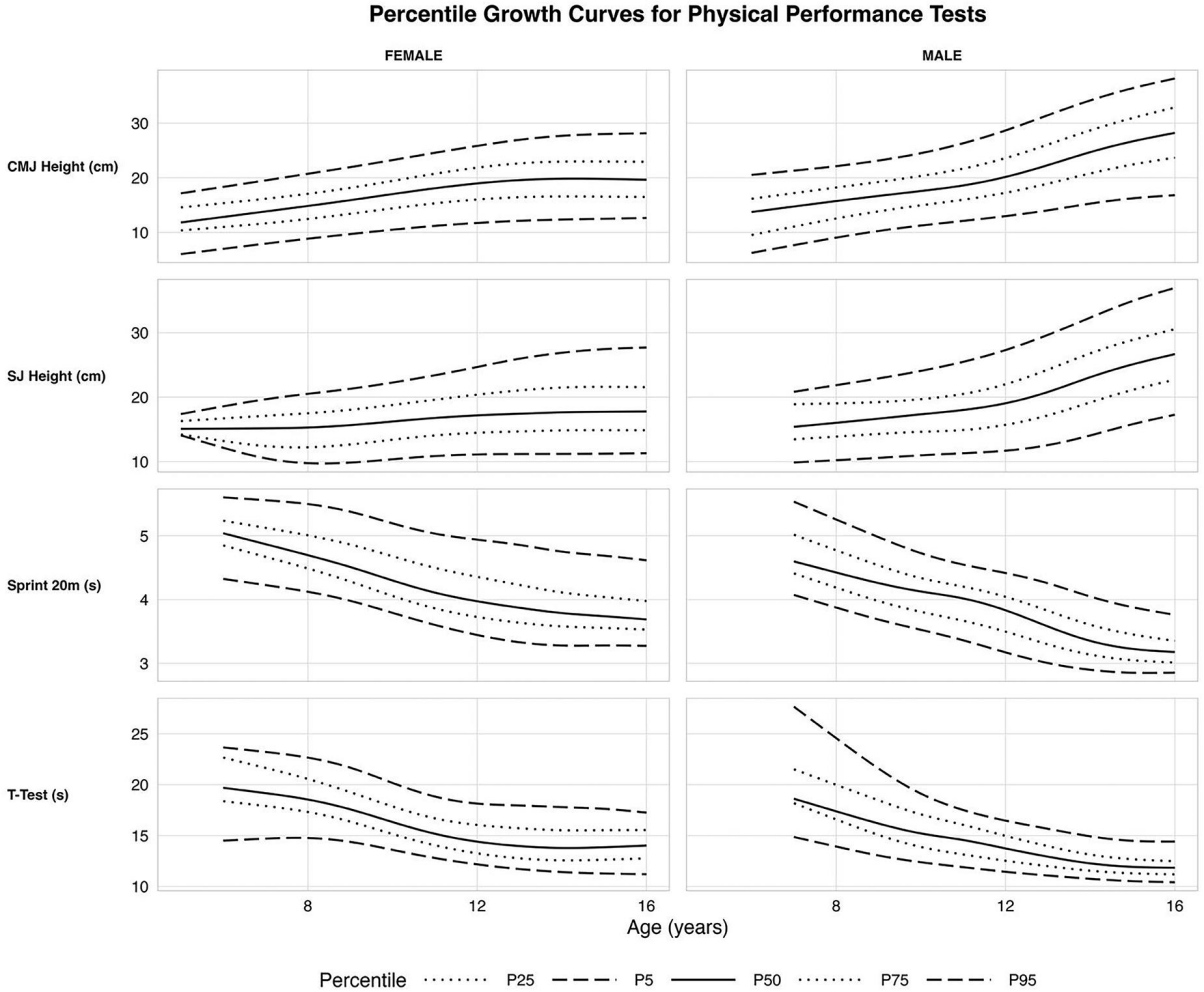
Linear regression models of physical performance as a function of age, stratified by sex. Scatter plots show individual performance data points for each metric (female: orange; male: blue). Best-fit linear regression lines illustrate the average annual rate of change in performance for each sex. Positive slopes indicate improvement in jump height; negative slopes indicate improvement in time-based metrics (lower times = better performance). All regression models were statistically significant (*p* < .001).

To quantify the linear relationship between age and performance, simple linear regression models were fitted for each metric, stratified by sex (Figure 4). All models confirmed significant associations between age and performance (all *p* < .001), with R^2^ values exceeding 0.60 across all tests, confirming that age is a strong predictor of physical performance in this school population. Regression slope coefficients indicate the average annual rate of improvement: CMJ height increased by 1.7 cm per year for males and 1.1 cm per year for females; sprint times decreased by 0.21 s per year for males and 0.16 s per year for females. These regression parameters provide school sport coordinators with practical, empirically grounded expectations for annual performance development, supporting realistic individual and collective goal-setting within the benchmarking framework.

## 4 Discussion

This longitudinal study analyzed physical performance in 2,473 school-aged students (ages 5–16) from Mexican private institutions, generating the first technology-derived normative dataset for this population using VALD Performance systems. Age was consistently associated with performance improvements across all five tests, sex-based differences expanded progressively during the adolescent years, and R^2^ values exceeded 0.60 across all regression models — supporting age as the primary stratification variable in the benchmarking framework. These findings address a genuine evidence gap in the Latin American sport science literature.

Age-related performance improvements were consistent with developmental data from European school populations. The greatest annual gains were observed between ages 13 and 14, consistent with normative data reported by Nowak et al. (7) in European youth football populations. CMJ height increased by 1.7 cm per year for males and 1.1 cm per year for females, comparable to rates reported by Taylor et al. (8) in English school populations. Males consistently outperformed females across all tests from approximately age 9–12 onward, consistent with Mascherini et al. (6). These trajectories provide school sport coordinators with empirically grounded expectations for annual development and programme evaluation.

Sex-based performance differences followed a consistent developmental pattern: small effects in early childhood expanding to large effects (Cohen's d > 0.8) from approximately age 14 for power-based tests and somewhat earlier for speed. This pattern is consistent with the differential developmental trajectories of lean muscle mass and neuromuscular capacity between sexes during adolescence (6, 8). Importantly, biological maturation was not assessed in this study, and the age-associated divergence should not be interpreted as a direct consequence of pubertal onset — future research should incorporate maturity offset calculations or Tanner staging to disentangle age and maturation effects (13).

### 4.1 Benchmarking framework for school sport management

The centile curves and normative tables generated in this study constitute the empirical foundation of a practical benchmarking system for school sport management (14, 15). Individual benchmarking is achieved by locating a student's result within the age- and sex-specific percentile distribution: P75 or above indicates an above-average profile for sport development, while P5 flags individuals warranting developmental support or health referral (12). Cohort-level benchmarking allows institutions to compare their mean performance profile against normative distributions to evaluate programme effectiveness. Longitudinal benchmarking (the most strategically valuable application) tracks individual percentile trajectories across annual assessments: maintained rank indicates expected development, while rank decline signals a relative deficit (1, 3). In physical education practice, these references support grade-specific development expectations, objective reporting to parents and administrators, and the systematic use of objective assessment to foster long-term engagement with physical activity among school-aged youth (16, 17). Future implementation should integrate this normative database into digital monitoring platforms for automated percentile tracking (18, 19).

### 4.2 Limitations and future directions

Several limitations should be acknowledged. First, biological maturation was not assessed: no anthropometric data were collected, precluding maturity offset calculations or developmental stage classification. Tounsi et al. (13) reported that sprint performance develops earlier relative to peak height velocity (PHV), whereas CMJ and SJ performance increases substantially around and after PHV due to increases in lower-limb muscle mass —highlighting the importance of incorporating maturation indicators in future studies. All references to pubertal timing in this manuscript are therefore contextual and not empirically derived from this dataset; future studies should incorporate validated maturation indicators such as the Mirwald equation or Tanner staging (20). Second, physical education quality, sport participation volume, and nutritional status were not measured, limiting attribution of observed performance levels to specific programmatic factors (21). Third, the sample is restricted to private schools in a single metropolitan area, limiting generalizability to public schools, rural settings, or other Mexican regions (22). Fourth, a substantial proportion of records represent cross-sectional rather than true repeated-measures observations; future research should implement prospective cohort designs with individual tracking (23). Finally, Ramirez-Campillo et al. (24) demonstrated that structured plyometric training substantially enhances power, speed, and agility in youth populations even with minimal intervention volume — suggesting that evidence-based programmes in this context could meaningfully shift the normative distributions reported here, a hypothesis warranting prospective investigation. Regarding talent identification, consistent with Fortin-Guichard et al. (25), objective physical metrics (particularly sprint time) are widely used for early talent detection; however, several authors (26, 27) caution against premature selection and instead recommend maximizing developmental opportunities for all youth athletes. Additionally, physical education exposure was not individually quantified, and sport participation outside school hours was not systematically controlled, which may have introduced variability in the neuromuscular development trajectories observed.

## 5 Conclusions

This longitudinal study establishes the first technology-validated normative dataset for physical performance in school-aged children and adolescents from Mexican private institutions. Age- and sex-specific centile curves and normative tables for five physical tests (CMJ, SJ, handgrip strength, 20-m sprint, and T-Test agility) provide a replicable benchmarking framework for school sport management, physical education planning, and talent identification.

Age was consistently associated with performance improvements across all physical capacities. Sex-based differences became more pronounced from approximately age 12, resulting in clearly differentiated trajectories that reinforce the importance of sex-specific normative references in school sport contexts. Ages 13–14 represent a critical developmental window and a priority period for periodized physical development interventions.

The benchmarking framework operationalised in this study enables evidence-based evaluation of program effectiveness, individual developmental tracking, and cohort-level institutional benchmarking. Although derived from private schools in a single metropolitan area, these normative values provide a foundation for broader national reference development. Future research should extend this framework to public schools and rural populations, incorporate biological maturation assessment, and implement prospective longitudinal tracking.

## Resource identification initiative

Statistical analyses were conducted using R (version 4.5.0; RRID: SCR_001905) in RStudio (RRID: SCR_000432).

## Data Availability

The original contributions presented in the study are included in the article/Supplementary Material, further inquiries can be directed to the corresponding author.
